# Discrepancy between Hepatitis C Virus Genotypes and NS4-Based Serotypes: Association with Their Subgenomic Sequences

**DOI:** 10.3390/ijms18010172

**Published:** 2017-01-17

**Authors:** Nan Nwe Win, Shingo Nakamoto, Tatsuo Kanda, Hiroki Takahashi, Azusa Takahashi-Nakaguchi, Shin Yasui, Masato Nakamura, Shuang Wu, Fumio Imazeki, Shigeru Mikami, Osamu Yokosuka, Tohru Gonoi, Hiroshi Shirasawa

**Affiliations:** 1 Department of Molecular Virology, Graduate School of Medicine, Chiba University, Chiba 260-8677, Japan; nannwewin@gmail.com (N.N.W.); sirasawa@faculty.chiba-u.jp (H.S.); 2 Department of Gastroenterology and Nephrology, Graduate School of Medicine, Chiba University, Chiba 260-8677, Japan; kandat-cib@umin.ac.jp (T.K.); ntcph863@yahoo.co.jp (S.Y.); nkmr.chiba@gmail.com (M.N.); gosyou100@yahoo.co.jp (S.W.); yokosukao@faculty.chiba-u.jp (O.Y.); 3 Medical Mycology Research Center, Chiba University, Chiba 260-8673, Japan; hiroki.takahashi@chiba-u.jp (H.T.); azusan_takahashi@faculty.chiba-u.jp (A.T.-N.); gonoi@faculty.chiba-u.jp (T.G.); 4 Safety and Health Organization, Chiba University, Chiba 263-8522, Japan; imazekif@faculty.chiba-u.jp; 5 Department of Gastroenterology, Kikkoman General Hospital, Noda 278-0005, Japan; smikami@mail.kikkoman.co.jp

**Keywords:** hepatitis C virus, discrepancy, genotypes, serotyping, genotyping

## Abstract

Determination of hepatitis C virus (HCV) genotypes plays an important role in the direct-acting agent era. Discrepancies between HCV genotyping and serotyping assays are occasionally observed. Eighteen samples with discrepant results between genotyping and serotyping methods were analyzed. HCV serotyping and genotyping were based on the HCV nonstructural 4 (NS4) region and 5′-untranslated region (5′-UTR), respectively. HCV core and NS4 regions were chosen to be sequenced and were compared with the genotyping and serotyping results. Deep sequencing was also performed for the corresponding HCV NS4 regions. Seventeen out of 18 discrepant samples could be sequenced by the Sanger method. Both HCV core and NS4 sequences were concordant with that of genotyping in the 5′-UTR in all 17 samples. In cloning analysis of the HCV NS4 region, there were several amino acid variations, but each sequence was much closer to the peptide with the same genotype. Deep sequencing revealed that minor clones with different subgenotypes existed in two of the 17 samples. Genotyping by genome amplification showed high consistency, while several false reactions were detected by serotyping. The deep sequencing method also provides accurate genotyping results and may be useful for analyzing discrepant cases. HCV genotyping should be correctly determined before antiviral treatment.

## 1. Introduction

Hepatitis C virus (HCV) is a positive-stranded RNA virus ~9.6 kb in length, and it belongs to the genus *Hepacivirus* within the *Flaviviridae* family [1]. It is estimated that 170 million people worldwide are chronically infected with HCV and at risk of liver fibrosis, cirrhosis and hepatocellular carcinoma [2]. The HCV genome encodes at least three structural proteins: core and two envelope glycoproteins (E1 and E2); and seven nonstructural (NS) proteins (p7, NS2, NS3, NS4A, NS4B, NS5A and NS5B).

Globally, at least seven HCV genotypes and 67 subtypes have been characterized. HCV isolates differ by >15% among different genotypes or subtypes [3]. HCV genotypes and subtypes are distributed differently among different areas of the world. In Japan, HCV genotype 1b is the major genotype (70%), followed by HCV genotype 2a (20%) and 2b (10%) [4,5]. Several methods have been established to determine HCV genotypes, such as sequence analysis [6], restriction fragment length polymorphism [7], hybridization of PCR products with specific probes [8] and next-generation sequencing [5].

Accurate determination of the HCV genotypes is essential for the selection of proper antiviral drugs and treatment regimens in HCV-infected individuals [9]. Treatment response also depends on HCV subgenotypes [10]. In Japan, the choice of antiviral regimen against HCV is generally based on the results of HCV serotyping as the national health insurance system currently approves only HCV serotyping methods [11]. Although previous reports indicate that the detection rate of serotyping is generally high and the risk of misdiagnosis is considered rare [11], we have experienced few cases with misidentified genotypes in the treatment of hepatitis C. It is important to elucidate the mechanism responsible for the discrepancy between HCV genotyping based on directly sequencing of the HCV genome and HCV serotyping by enzyme immunoassay (EIA).

In the present study, we described cases with discrepant results between HCV genotyping and serotyping assays that were based on the 5′-untranslated region (5′-UTR) and NS4 regions, respectively. Sanger sequencing was performed for the HCV core and NS4 regions, and deep sequencing was conducted for HCV NS4 regions to study the validity of the serotyping and genotyping results.

## 2. Results

### 2.1. Patients’ Characteristics

In the present study, the patients consisted of 11 males and seven females with a mean age of 62.2 years (Table 1). Among these 18 patients, histories of multiple intravenous injections, tattooing and blood transfusion were observed in 4, 5 and 2 patients, respectively. Family histories of liver diseases existed in four patients, of whom three were anti-HCV positive. Only one patient (No. 18) had very low HCV RNA (<1.2 logIU/mL).

### 2.2. HCV Genotyping and HCV Serotyping

Among 18 HCV isolates, HCV genotyping assays judged 4, 5 and 9 isolates as HCV genotypes 1b, 2a and 2b, respectively (Table 2). Results of HCV serotyping are also shown in Table 2. Among four HCV genotype 1b isolates, 2, 1 and 1 were judged as HCV serotype 2, not determined and mixed, respectively. Among five HCV genotype 2a isolates, 3, 1 and 1 were judged as HCV serotype 1, not determined and mixed, respectively. Among nine HCV genotype 2b isolates, six and two were judged as HCV serotype 1 and not determined, respectively. For No. 17, genotype could not be determined by the type-specific PCR method in the core region [12], thus included in the study. This sample was determined as serotype 2, consistent with the sequencing results of genotype 2b.

### 2.3. Sanger Sequencing Results of Clones from HCV Core and NS4 Regions

Table 2 shows the Sanger sequencing results in the core and NS4 regions, respectively. In 17 of 18 samples, both results of cloned sequencing in the core and NS4 regions were consistent with the HCV genotyping by 5′-UTR, but not with those of HCV serotyping. PCR amplification for one isolate (No. 18) could not be performed for core and NS4 regions due to the low titer of HCV RNA. Nucleotide sequences of the HCV core region obtained by sequencing were compared with those of reference sequences (Table 3). Nucleotide sequences and amino acid sequences of HCV NS4 regions were also compared with those of reference sequences (Table 4 and Table 5). Sequence identity was generally high within the same subgenotype and higher in the core region than in the NS4 region.

### 2.4. Cloning Analysis of NS4 Epitope Regions

Epitope regions of NS4 antigen were described previously [14]. To examine whether the variations of NS4 epitope regions could affect the enzyme immunoassay results, amino acids of epitope regions in each clone were compared with those of group specific peptides 1 and 2 (Table 5B,C). Except for a few variations between amino acid sequences of the five clones, most of the sequences were the same within each sample. In comparison with the reference peptide, there were several amino acid variations in each sample except in No. 8. However, only a few cases had same amino acid as the reference of different genotypes (underlined in Table 5B,C), and each sequence was much closer to the reference peptide with the same genotype.

### 2.5. Results of HCV Genotyping by Deep Sequencing in NS4 Regions

**Figure 1:**
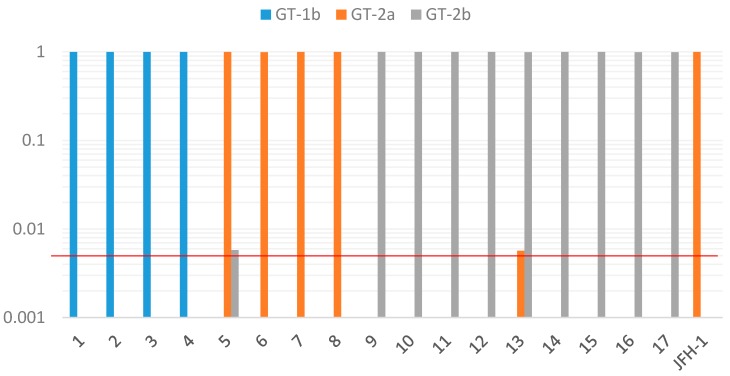
HCV genotypes (GTs) by deep sequencing of HCV NS4 regions. For each sample, only populations that exist at a frequency of >0.005 (red line) were shown by log-scale. No. 5 with the main genotype 2a had minor populations assigned to the different genotype 2b. No. 13 with genotype 2b had minor populations assigned to the different genotype 2a.

Genotyping results investigated by deep sequencing of HCV NS4 regions using the MiSeq Illumina sequencing method are shown in Figure 1 and Table 6. For all 17 samples, more than 99% of the sequence reads were assigned to the same genotype, as with the genotyping results by other methods. The results were quite similar when different sequencing kits were compared between the nano kit and standard kit, within the same samples (No. 2, 7, 17) (data not shown). Among the 17 samples, genotype 2a (No. 5) and genotype 2b (No. 13) had minor populations assigned to different genotypes (genotype 2b and 2a, respectively) from major populations and were considered as mixed infection. The prevalence of these minor populations was between 0.5% and 1% (457 reads (0.58%) for Sample 5 and 5010 reads (0.57%) for Sample 13). These minor populations are unlikely to be related to the serotyping results, because serotype 1 was detected from both samples (Table 2).

## 3. Discussion

The distribution of HCV genotypes is different among individuals with different infection routes such as blood transfusion, intravenous drug use and tattooing. In Japan, HCV genotype 1b had been associated with blood transfusion, whereas genotypes 2a and 2b were associated with injections or tattooing [15]. In the present study, 14 of 18 patients were confirmed as HCV genotype 2, supporting the previous report that described that the NS4-based serotyping assay had lower concordance for genotype 2b specimens [16]. Among these 14 patients, seven (50%) had a history of intravenous injection or tattooing. These patients are considered to be multiple HCV-exposed individuals, and some of them might have been exposed to mixed infection with different HCV genotypes and subtypes.

In the present study, the same genotype was detected by cloned Sanger sequencing at the core and NS4 regions of the HCV genome as the genotype determined by 5′-UTR. No case was detected as mixed infection. As the possibility of the coexistence of a few minor variants could not be completely ruled out because only five clones were analyzed in this study, we further examined this possibility by the deep sequencing method. Deep sequencing of the HCV NS4 region produced large sequence depth ranging from 45 to 993 thousands of reads from each sample. In all 17 cases of the present study, more than 99% of the reads were assigned to the same genotype as determined by other genotyping methods. Furthermore, in two of the 17 samples, a minor population of sequences with different HCV subgenotypes was detected, suggesting the possibility of mixed infection in these samples. It is difficult to detect minor variants, which is between 0.5% and 1%, by the standard cloning method. Deep sequencing could rapidly and easily detect minor variants with a large sequence depth compared to the cloning method. As for sequencing error, our study using the JFH1 clone revealed that up to 0.4% of reads were assigned to incorrect genotype (2b), consistent with a previous report describing a sequencing error rate of below 0.4% in the Illumina platform [17]. However, Thomson et al. [18] suggested that the detection of minority variants is less reliable at lower ratios. The cases of mixed infection with different HCV genotypes, as well as the recombinant forms of HCV are very rare, even in a highly exposed group, such as intravenous drug users (IVDU) [19].

Among the 18 cases, 11 were confirmed to show different HCV serotyping results from HCV genotyping; two cases showed HCV genotype 1b with HCV serotype 2; while nine cases showed HCV genotype 2 with HCV serotype 1. Compared to the core region, nucleotide sequence variations in the NS4 region were more commonly existing between samples within the same genotype (Table 3 and Table 4). We examined the possibility that variations of the amino acid sequence could affect the serotyping results. Both consensus sequence (Table 5A) and cloning analysis in the NS4 region (Table 5B,C) showed that although several amino acid variations existed when compared with the HCV genotype-specific reference peptide, each sequence was much closer to the reference peptide with the same genotype. It should be noted that the error rate of Taq DNA polymerase is approximately 2 × 10^−5^, estimating that one nucleotide error could appear in 50% of the amplified DNA molecules by nested PCR. Furthermore, comparative analysis with the control group will be needed to better understand the mechanism of discrepancy.

Among the 18 HCV specimens serotyped in our series, four samples (22.2%) were defined as “not determined”. It has been suggested that HCV serotyping analysis provides an indirect typing method based on the production of type-specific antibodies by the infected host. Therefore, the sensitivity of HCV serotyping depends on the immune response of the HCV-infected host. Therefore, the possibility that some of the patients with HCV infection did not produce antibody against NS4 protein should be considered. By the HCV serotyping assay, two of the 18 patients (11.1%) had mixed reactions. Multiple exposures to different HCV strains could be one possible mechanism for these findings, although the possibility of non-specific reaction also exists. For such cases, instead of HCV serotyping, HCV genotype testing can be a good alternative method.

A major limitation of our study is that we could not describe the sensitivity and concordance between HCV serotyping and genotyping data in the entire cohort due to lack of a comparable group. Previous studies [11,20] using the same NS4 antigen (C14) as ours showed that the sensitivity of serotypes 1 and 2 is 95.8%–100%, and there was no discordant case. We repeated the initial serotyping assay using the same antigen with the newly-updated chemiluminescent EIA system (HISCL HCV Gr reagents, Sysmex, Japan). According to the manufacturer’s instruction, the sensitivity of the new system is 84.4%, and the serotyping results showed 100% concordance with those of the older system. Our re-serotyping analysis showed that new system could correctly detect type 2-specific antibody in six samples (Nos. 6, 8, 10, 12, 17 and 18) and type 1 antibody in two samples (Nos. 2 and 4). However, it could not detect any antibodies in eight cases, showed the incorrect serotype in one case (No. 14) and mixed serotype in one case (No. 11). These results suggest that comparing different serotyping systems would be useful to understand the discrepant cases further.

After the identification of serotype-specific linear epitopes in the core and NS4 region [11,14], several serotyping systems have been reported using peptides corresponding to these regions. Besides the C14 serotyping system we used, the MUREX HCV Serotyping 1–6 assay (Abbott Diagnostics) has been developed [21] using type-specific NS4 antigen corresponding to HCV types 1–6. Prescott et al. [22] reported that 15 discrepant cases (151/166) could be detected using the old version of this assay (HC02). According to the instructions, the concordance (96.12%) of the new version (2G26) becomes higher than an earlier version (HC02) (90.37%), while the sensitivity of serotypes 1 and 2 is 76.8% and 75.5% in the new version. The RIBA HCV serotyping assay (Chiron Corporation) [16,23,24] detected anti-HCV antibodies against five synthetic peptides from the NS4 region and three peptides from the core region, and it has been reported that the sensitivity/concordance of serotypes 1 and 2 is 84.7%–96.5%/96.2%–100% and 82.4%–100%/93.4%–100%, respectively. Taken together, serotyping generally shows high concordance with genotyping. However, attention should be paid to the fact that there could occur cases with no response to the antigen and also with discrepancy even if they can be regarded as uncommon events based on previous studies.

Only sequence analysis of specific gene regions of the HCV genome that are predictive of HCV genotype was completely reliable for HCV genotyping [25]. There are several genotyping methodologies available, such as sequencing, primer-specific PCR, real-time PCR and the line probe assay. Chantratita et al. [26] compared two PCR-based line probe assays and recently reported that the six HCV genotyping 9G test showed overall sensitivity and specificity higher than 92.5% and 99.4%, respectively. On the other hand, this method based on PCR may not be correctly estimated in cases with low-level viremia, such as No. 18 in this study, or if serum samples have not been stored correctly [27]. Meanwhile, one sample (No. 17) could not be genotyped by the HCV genotype-specific PCR method. In this case, even small sequence variations in the primer region could affect the results due to high sequence similarity in this region (Table 3).

Serological methods are based on the detection of antibodies against HCV serotype-specific epitopes and are easier and faster for determining HCV serotypes. It is possible that HCV serotyping may allow the determination of HCV types of both past and present infection. Moreover, its high performance on samples with low viral load offers an obvious advantage over the genotyping methods. The cost of the HCV serotyping method is less than that of HCV genotyping. ELISA of the HCV serotyping method is seldom associated with the risk of contamination. Thus, HCV serotyping is particularly suitable for epidemiologic studies.

Treatment outcomes of patients are shown in Table 7. In two of four patients with HCV genotype 1b, treatment with daclatasvir plus asunaprevir for 24 weeks could lead to sustained virological response at 24 weeks after the stoppage of treatment (SVR24). In four of five patients with HCV genotype 2a, treatment with sofosbuvir plus ribavirin was performed for 12 weeks, and all four patients achieved SVR24. Among the total of nine patients with HCV genotype 2b, five were treated with interferon-free or interferon-including regimens and achieved SVR24. Although six patients (Nos. 1, 2, 3, 4, 9, 16) had been treated with interferon-including treatments without DAAs based on serotyping results, some patients (Nos. 2, 4, 9), developed relapse after completing the treatment course, and others (Nos. 1, 3, 16) had no response.

Better treatment outcome can be obtained when the treatment is based on HCV genotyping results. For example, Patient No. 2 had been a candidate for sofosbuvir and ribavirin regimen based on the serotyping results (serotype 2), but the patient was treated with daclatasvir and asunaprevir regimen after getting the genotyping result of genotype 1b and achieved SVR24. Sohda et al. [28] reported the non-response to daclatasvir and asunaprevir therapy in patients coinfected with hepatitis C virus genotypes 1 and 2.

## 4. Patients and Methods

### 4.1. Study Population

Sera were obtained from 18 Japanese patients (11 males and 7 females) with chronic HCV infection and were stored at −20 °C until testing at Chiba University, Graduate School of Medicine. These patients had been previously serotyped when the direct-acting antiviral agents (DAAs) were not available. They were genotyped at the time of DAA treatment; however, discrepant results between HCV genotyping and serotyping were observed in all of these samples. All specimens were HCV RNA-positive by the Taqman reverse transcriptase-polymerase chain reaction (RT-PCR) assay (Roche Diagnostics, Tokyo, Japan), with levels ranging from 1.2 to 7.5 log IU/mL, and were negative for HBsAg or anti-HIV by ELISA. There was no sign of hepatocellular carcinoma in any of the patients, although Patient Nos. 7 and 14 had histories of cirrhosis. More clinical information is described in Table 1. This study was approved by ethics committee of Chiba University, Graduate School of Medicine (No. 415/1753/2153). Participation in the study was posted at our institutions.

### 4.2. HCV Genotyping and Serotyping Assays

HCV genotyping was performed by RT-PCR followed by Sanger direct sequencing at 5′-UTR of the HCV genome, except Sample No. 17, which was determined by the type-specific PCR method in the core region [12]. HCV serotyping was determined by detecting antibodies against group-specific recombinant protein for serotypes 1 and 2 in the putative HCV NS4 protein region by an EIA that is commonly used in Japan [11]. According to this assay, HCV serotypes 1 and 2 correspond to HCV genotypes 1a/1b and 2a/2b, respectively. The results were described as “not determined” when no antibody could be detected and as “mixed” when both antigens reacted with serum samples.

### 4.3. HCV RNA Extraction and RT-PCR

Briefly, total RNA was extracted from 200 μL of sera by the High Pure Viral RNA Kit (Roche, Mannheim, Germany). cDNA was synthesized from viral RNA using SuperScript III First-Strand Synthesis SuperMix (Invitrogen, Carlsbad, CA, USA) according to the manufacturer’s instructions. The presence of HCV RNA in serum was confirmed by nested RT-PCR using primers of the 5′-UTR-core; Sc2 (sense; 5′-GGGAGGTCTCGTAGACCGTGCACCATG-3′, nucleotide position 318–344) and Ac2 (antisense; 5′-GAGMGGKATRTACCCCATGAGRTCGGC-3′, 758–732) [12] and primers of NS4 regions; 5668 (sense; 5′-ATGCATGTCRGCTGAYCTGGA-3′, 5282–5302) and 007 (antisense; 5′-AACTCGAGTATCCCACTGATGAAGTTCCACAT-3′, 5665–5634) [22] in the first round. Two microliters of cDNA were amplified for 40 cycles with the following parameters: a preliminary 20 cycles of amplification at 94 °C for 1 min (denaturing), 45 °C for 1 min (annealing) and 72 °C for 1 min (extension), followed by 20 additional cycles at 94 °C for 1 min, 55 °C for 1 min and 72 °C for 1 min using the HotStarTaq Master Mix Kit (QIAGEN, Hilden, Germany). For second-round PCR, 2 μL of first-round PCR product were amplified for 35 cycles; each cycle consisted of 94 °C for 1 min, 55 °C for 1 min and 72 °C for 1 min using HotStarTaq Master Mix Kit (QIAGEN) with primers of the 5′-UTR-core; S7 (sense; 5′-AGACCGTGCACCATGAGCAC-3′, 330–349) and A5 (antisense; 5′-TACGCCGGGGGTCAKTRGGGCCCCA-3′, 684–660) [12] or those of NS4 regions; 865 (sense; 5′-CTGGAGGTTATCACNAGCACNTGG-3′, 5298–5321) and 220 (antisense; 5′-CACATGTGCTTCGCCCAGAA-3′, 5638–5619) [22]. Nucleotide positions are described according to the sequence H77 (Accession No. AF009606). Two rounds of amplification were performed on TaKaRa PCR Thermal Cycler Dice (Takara, Otsu, Japan).

### 4.4. Cloning and Sanger Sequencing

Cloning of each PCR product into the TOPO vector (Invitrogen) was carried out using the TOPO TA Cloning Kit (Invitrogen) according to the manufacturer’s instructions. Up to 5 different clones were picked up for sequencing. Inserted fragments were confirmed by colony PCR using M13 primers (sense, 5′-GTAAAACGACGGCCAG-3′, and antisense, 5′-CAGGAAACAGCTATGAC-3′) with the following conditions: amplification for 30 cycles at 94 °C for 1 min, 55 °C for 1 min and 72 °C for 1 min.

PCR products were purified by the QIAquick PCR Purification Kit (QIAGEN), and submitted to Sanger DNA sequencing using an ABI3730XL DNA analyzer (Applied Biosystems, Waltham, MA, USA). Obtained sequences were aligned and phylogenetic analysis was performed using Genetyx software (Genetyx Corp., Tokyo, Japan). Reference sequences for each genotype were used as follows: AF009606 (H77) for genotype 1a; D10934 (HC-C2), D90208 (HCV-J) and AJ238799 (Con1) for HCV genotype 1b; AB047639 (JFH-1) and D00944 (HC-J6) for HCV genotype 2a; and AY232731 (MD2b1-2) and D10988 (HC-J8) for HCV genotype 2b [13]. The consensus sequence for each sample was determined from the cloned sequence data. The sequences determined in this study have been deposited in the GenBank database (Accession Nos. LC131493–LC131642, LC191872–LC191891).

### 4.5. Deep Sequencing

The MiSeq platform (Illumina, San Diego, CA, USA) was used to analyze the HCV NS4 region, which was amplified by nested PCR using adaptor primers (sense, 5′-TCGTCGGCAGCGTCAGATGTGTATAAGAGACAGCTGGAGGTTATCACNAGCACNTGG-3′, and antisense, 5′-GTCTCGTGGGCTCGGAGATGTGTATAAGAGACAGCACATGTGCTTCGCCCAGAA-3′) with a KAPA HiFi HotStart ReadyMix PCR kit (KAPA BIO, Boston, MA, USA). PCR for the first round was 5 min at 95 °C; followed by 30 cycles for 30 s at 95 °C, 30 s at 65 °C and 30 s at 72 °C. Purified PCR products were amplified using different sets of index primers in the Nextera XT Index kit (Illumina) for each sample. The PCR conditions for the amplification were 95 °C for 3 min, followed by 8 cycles of 95 °C for 30 s, 55 °C for 30 s and 72 °C for 30 s with a final extension of 72 °C for 5 min. After purification, the products were quantified using the Quant-iT PicoGreen double-stranded DNA (dsDNA) Reagent and Kit (Invitrogen) according to the manufacturer’s instructions. Paired-end sequencing was conducted on the Illumina MiSeq platform using a MiSeq Reagent Kit Nano v2 500 cycles (Illumina) according to the manufacturer’s protocol. A MiSeq v2 Reagent Kit 500 cycles (Illumina) was used in Nos. 13, 14 and JFH-1 for control.

### 4.6. Data Analysis of Deep Sequencing

After filtering by PANDAseq (v2.8) with the parameters “-L 300 -L 200” [29], an average of 70,157 reads was produced from each sample by the Nano kit while an average of 937,428 reads was produced by the standard kit. Each read was aligned against reference HCV genomes (H77, HCV-J, HC-J6 and HC-J8) using BLASTN (v2.2.28+) with the parameters “-evalue 1e-4–task blastn” [30]. Reference sequences with non-identical genotypes to samples were also included (D17763 (NZL1) for HCV genotype 3a, Y11604 (ED43) for 4a, Y13184 (EUH1480) for 5a, Y12083 (EUHK2) for 6a and EF108306 (QC69) for 7a, respectively) [13]. Mixed infection was identified by the generation of multiple contigs against two or more reference genomes. For control experiments, HCV RNA was prepared from conditioned medium on Huh7 cells transfected with HCV JFH1 [1] and was subjected to deep sequencing. Among 994,646 hit reads to the references, 990,097 reads (99.54%) were correctly assigned to the original JFH1 sequences. When HC-J6 was used as a reference of genotype 2a, 988,099 (99.42%) were correctly assigned to genotype 2a, while 4058 reads (0.41%) were assigned as genotype 2b, which were regarded as sequencing errors. Based on these data, the threshold for the detection of minor populations with different genotypes was set at 0.5%.

## 5. Conclusions

Although the HCV serotyping method is a widely applicable method especially when many samples are to be tested, there are some discrepant cases, as well. Currently, there are several serotyping systems with high accuracy available. Using the latest system and combining different methods should minimize the risk of discrepancy. Rapid genotyping systems can be a better alternative to serotyping, although it is still costly. The present study highlighted ultra-deep sequencing, and it may be useful for the diagnosis of patients with mixed HCV genotypes. Until the availability of effective antiviral regimens for the pangenotypes of HCV, HCV genotyping should be correctly determined before antiviral therapy, especially in certain regimens, for which the efficacy is dependent on HCV genotypes.

## Figures and Tables

**Table 1:** Background of patients enrolled in the present study.

No.	Age/Sex	Risk Factors	Family Histories of Liver Diseases	HCV RNA (Log IU/mL)	ALT (IU/L)	PLT (10^4^/μL)	ALB (g/dL)	Total Bilirubin (mg/dL)	AFP (ng/mL)
1.	56/F	Unknown	−	6.6	52	19.6	4.1	0.5	20.2
2.	71/M	None	+	7.5	61	12	4.6	1.3	2.3
3.	66/M	Tattoo	−	5.9	52	9.3	3.8	1.0	8.1
4.	51/M	Blood transfusion	−	6.7	53	26.2	4.5	0.5	6.1
5.	61/F	None	+	>5.9	14	18.7	4.1	0.9	3.4
6.	81/M	None	+	6	253	12.3	4.3	0.6	10.4
7.	74/M	IVDU	−	6.4	62	9.1	3.7	0.9	11.5
8.	57/F	Unknown	−	>5.9	17	21.3	4.4	0.5	6
9.	50/M	IVDU	−	7.2	24	15.4	4.3	0.7	3
10.	47/M	Tattoo and IVDU	−	6.8	52	18.5	4	0.5	2.6
11.	58/F	Tattoo	−	>5.9	22	19.3	4.8	1.1	2.7
12.	65/F	Tattoo	−	7	18	22.3	4.7	0.7	2.2
13.	71/F	None	−	6.2	16	17.3	4.2	0.6	2.6
14.	62/M	Blood transfusion	−	6.4	21	15.7	4.6	0.4	5.1
15.	58/M	Unknown	−	NA	23	13.9	3.4	0.3	3.4
16.	62/F	None	+	6	38	19.5	4.3	0.6	2.5
17.	57/M	Tattoo	−	4.4	130	13.7	4.1	0.7	6
18.	72/M	IVDU	−	<1.2	21	13.2	4.1	0.5	3.6

No., patient number; HCV, hepatitis C virus; ALT, alanine transaminase; PLT, platelet counts; ALB, albumin; AFP, α-fetoprotein; M, male; F, female; −, negative; +, positive; NA, not available.

**Table 2:** Comparison between the results of serotyping, genotyping, Sanger sequencing and deep sequencing methods for discrepant samples.

No.	Serotyping	Genotyping (5′-UTR)	Genotyping by Cloned Sanger Sequencing		Genotyping by Deep Sequencing (NS4)
			Core	NS4	
1.	2	1b	1b	1b	1b
2.	2	1b	1b	1b	1b
3.	ND	1b	1b	1b	1b
4.	Mixed	1b	1b	1b	1b
5.	1	2a	2a	2a	2a + 2b *
6.	1	2a	2a	2a	2a
7.	1	2a	2a	2a	2a
8.	ND	2a	2a	2a	2a
9.	1	2b	2b	2b	2b
10.	1	2b	2b	2b	2b
11.	1	2b	2b	2b	2b
12.	1	2b	2b	2b	2b
13.	1	2b	2b	2b	2a * + 2b
14.	1	2b	2b	2b	2b
15.	ND	2b	2b	2b	2b
16.	ND	2b	2b	2b	2b
17.	2	ND **	2b	2b	2b
18.	Mixed	2a	ND	ND	ND

ND, not determined; Mixed, co-reaction to serotypes 1 and 2. * Minor different genotypes; ** this sample was included in the study due to the failure for genotyping by type-specific PCR method in the core region, but later found as genotype 2b by sequence-based genotyping.

**Table:** (**A**)

Ref.	H77	HC-C2	HCV-J	Con1	No. 1	No. 2	No. 3	No. 4
H77	100% (309/309)							
HC-C2	92.6% (286/309)	100% (309/309)						
HCV-J	92.2% (285/309)	97.1% (300/309)	100% (309/309)					
Con1	92.2% (285/309)	97.4% (301/309)	97.1% (300/309)	100% (309/309)				
No. 1	91.8% (1419/1545)	97.4% (1505/1545)	96.7% (1494/1545)	97.8% (1511/1545)	98.9% (4585/4635)			
No. 2	90.8% (1403/1545)	96.6% (1493/1545)	97.0% (1498/1545)	96.0% (1483/1545)	95.9% (7405/7725)	99.8% (4627/4635)		
No. 3	91.8% (1419/1545)	97.7% (1509/1545)	98.1% (1516/1545)	97.2% (1501/1545)	97.0% (7495/7725)	96.9% (7485/7725)	99.5% (4613/4635)	
No. 4	92.3% (1426/1545)	96.6% (1492/1545)	97.5% (1506/1545)	97.2% (1502/1545)	96.8% (7480/7725)	96.4% (7450/7725)	97.4% (7525/7725)	99.7% (4619/4635)

Reference sequences for each genotype were used as follows: H77 for HCV genotype 1a; HC-C2, HCV-J and Con1 for HCV genotype 1b; JFH-1 and HC-J6 for HCV genotype 2a; MD2b1-2 and HC-J8 for HCV genotype 2b [13].

**Table:** (**B**)

Ref.	JFH-1	HC-J6	No. 5	No. 6	No. 7	No. 8
JFH-1	100% (309/309)					
HC-J6	94.5% (292/309)	100% (309/309)				
No. 5	94.5% (1460/1545)	96.1% (1486/1545)	98.8% (4581/4635)			
No. 6	94.6% (1461/1545)	96.8% (1496/1545)	95.3% (7365/7725)	99.7% (4621/4635)		
No. 7	93.3% (1442/1545)	95.6% (1477/1545)	95.0% (7339/7725)	94.4% (7290/7725)	97.4% (4515/4635)	
No. 8	94.8% (1465/1545)	96.8% (1496/1545)	96.1% (7424/7725)	95.9% (7405/7725)	94.8% (7324/7725)	99.4% (4605/4635)

**Table:** (**C**)

Ref.	HC-J8	MD2b1-2	No. 9	No. 10	No. 11	No. 12	No. 13	No. 14	No. 15	No. 16	No. 17
HC-J8	100% (309/309)										
MD2b1-2	97.4% (301/309)	100% (309/309)									
No. 9	95.6% (1477/1545)	95.6% (1477/1545)	99.6% (4615/4635)								
No. 10	96.9% (1497/1545)	97.4% (1505/1545)	95.5% (7375/7725)	98.7% (4574/4635)							
No. 11	96.0% (1483/1545)	96.1% (1485/1545)	94.6% (7310/7725)	96.6% (7466/7725)	98.6% (4572/4635)						
No. 12	96.5% (1491/1545)	97.2% (1501/1545)	96.3% (7439/7725)	97.5% (7535/7725)	96.5% (7452/7725)	98.8% (4581/4635)					
No. 13	95.5% (1475/1545)	95.7% (1479/1545)	93.7% (7328/7725)	94.5% (7300/7725)	94.3% (7285/7725)	94.7% (7316/7725)	97.7% (4528/4635)				
No. 14	97.5% (1506/1545)	97.5% (1506/1545)	95.8% (7400/7725)	96.8% (7478/7725)	96.0% (7416/7725)	96.8% (7478/7725)	95.5% (7377/7725)	99.6% (4616/4635)			
No. 15	96.4% (1489/1545)	96.0% (1483/1545)	95.5% (7375/7725)	95.8% (7400/7725)	95.4% (7372/7725)	96.0% (7413/7725)	94.8% (7323/7725)	95.8% (7400/7725)	99.2% (4598/4635)		
No. 16	95.0% (1468/1545)	95.5% (1476/1545)	95.2% (7352/7725)	95.5% (7381/7725)	94.8% (7325/7725)	95.6% (7384/7725)	93.8% (7246/7725)	95.2% (7354/7725)	95.0% (7340/7725)	99.1% (4591/4635)	
No. 17	94.6% (1462/1545)	95.3% (1472/1545)	94.2% (7280/7725)	94.8% (7320/7725)	93.3% (7205/7725)	94.8% (7320/7725)	93.1% (7192/7725)	94.7% (7316/7725)	94.1% (7270/7725)	94.0% (7265/7725)	99.4% (4609/4635)

**Table:** (**A**)

Ref.	H77	HC-C2	HCV-J	Con1	No. 1	No. 2	No. 3	No. 4
H77	100% (297/297)							
HC-C2	76.1% (226/297)	100% (297/297)						
HCV-J	78.1% (232/297)	90.9% (270/297)	100% (297/297)					
Con1	77.1% (229/297)	88.9% (264/297)	90.9% (270/297)	100% (297/297)				
No. 1	77.3% (1148/1485)	89.4% (1328/1485)	94.3% (1400/1485)	91.0% (1352/1485)	98.2% (4376/4455)			
No. 2	76.4% (1134/1485)	88.8% (1319/1485)	89.6% (1331/1485)	87.8% (1304/1485)	88.7% (6585/7425)	99.9% (4451/4455)		
No. 3	74.7% (1110/1485)	90.9% (1350/1485)	89.4% (1328/1485)	90.4% (1343/1485)	88.9% (6601/7425)	88.5% (6570/7425)	99.2% (4421/4455)	
No. 4	77.5% (1151/1485)	92.3% (1370/1485)	92.3% (1370/1485)	92.3% (1371/1485)	91.9% (6825/7425)	91.6% (6800/7425)	93.5% (6940/7425)	99.7% (4441/4455)

**Table:** (**B**)

Ref.	JFH-1	HC-J6	No. 5	No. 6	No. 7	No. 8
JFH-1	100% (297/297)					
HC-J6	88.6% (263/297)	100% (297/297)				
No. 5	89.3% (1326/1485)	91.6% (1361/1485)	98.5% (4389/4455)			
No. 6	91.2% (1355/1485)	91.7% (1362/1485)	94.0% (6980/7425)	98.5% (4389/4455)		
No. 7	87.8% (1304/1485)	90.8% (1349/1485)	92.3% (6850/7425)	90.9% (6748/7425)	96.6% (4304/4455)	
No. 8	90.6% (1346/1485)	92.1% (1368/1485)	92.3% (6854/7425)	90.7% (6735/7425)	91.2% (6771/7425)	99.2% (4419/4455)

**Table:** (**C**)

Ref.	HC-J8	MD2b1-2	No. 9	No. 10	No. 11	No. 12	No. 13	No. 14	No. 15	No. 16	No. 17
HC-J8	100% (297/297)										
MD2b1-2	94.6% (281/297)	100% (297/297)									
No. 9	92.4% (1372/1485)	89.7% (1332/1485)	99.7% (4442/4455)								
No. 10	91.9% (1365/1485)	93.1% (1383/1485)	88.8% (6590/7425)	98.7% (4397/4455)							
No. 11	93.9% (1395/1485)	93.9% (1395/1485)	89.8% (6665/7425)	93.3% (6925/7425)	98.9% (4407/4455)						
No. 12	95.0% (1411/1485)	95.9% (1424/1485)	90.4% (6710/7425)	94.1% (6984/7425)	95.3% (7073/7425)	98.9% (4407/4455)					
No. 13	92.4% (1372/1485)	92.1% (1368/1485)	89.9% (6675/7425)	91.8% (6816/7425)	92.7% (6883/7425)	93.2% (6920/7425)	99.8% (4446/4455)				
No. 14	92.8% (1378/1485)	92.9% (1380/1485)	89.3% (6631/7425)	93.1% (6913/7425)	93.7% (6957/7425)	93.9% (6972/7425)	93.3% (6928/7425)	99.2% (4419/4455)			
No. 15	92.9% (1380/1485)	91.6% (1360/1485)	90.5% (6720/7425)	92.3% (6856/7425)	92.7% (6885/7425)	93.8% (6965/7425)	91.3% (6779/7425)	92.7% (6883/7425)	99.4% (4427/4455)		
No. 16	92.0% (1366/1485)	90.6% (1346/1485)	90.2% (6695/7425)	90.6% (6726/7425)	91.1% (6765/7425)	91.7% (6810/7425)	92.4% (6861/7425)	91.3% (6779/7425)	90.8% (6740/7425)	99.3% (4423/4455)	
No. 17	89.6% (1330/1485)	87.2% (1295/1485)	89.7% (6660/7425)	85.5% (6351/7425)	87.3% (6484/7425)	88.2% (6551/7425)	89.4% (6638/7425)	86.3% (6408/7425)	86.3% (6406/7425)	88.1% (6540/7425)	98.2% (4375/4455)

**Table:** (**A**)

Group 1 Peptide	FTTGSVVIVGRIILSGRPAVIPDREVLYREFDEMEECASHLPYIEQGMQLAEQFKQKALGLLQTATKHAEAAAPVVESKWRALET
HC-C2	L...........V...............Q........G.........................I...Q.................
HCV-J	L...........................Q......................................Q................V
Con1	L...............K..I......................................I........Q.............T..A
No. 1	L...............K...........Q......................................Q............Q...A
No. 2	L...................V................T.............................Q......M.....Q...A
No. 3	L..................................................................Q.................
No. 4	L...............K..................................................Q.................
Group 2 peptide	.A..C.S.I..LHINQ.AV.A..K....EA.........RAAL..E.QRI..ML.S.IQ....Q.S.Q.QDIK.A.QTS.PKV.Q
JFH-1	LA..C.S.I..LHVNQ.VV.A..K....EA.........RAAL..E.QRI..ML.S.IQ....Q.S.Q.QDIQ.AMQAS.PKV.Q
HC-J6	LA..C.C.I..LHVNQ.AV.A..K....EA.........RAAL..E.QRI..ML.S.IQ....Q.S.Q.QDIQ.A.QAS.PKV.Q
No. 5	LA..C.S.I..LHINQ.AV.A..K....EA.........KAAL..E.QRI..ML.S.IQ....Q.S.Q.QDIQ.A.QAS.PKV.Q
No. 6	LA..CVT.I..LHVNQ.AV.A..K....EA.........KAAL..E.QRI..ML.S.IQ....Q.S.Q.QDIQ.A.QAS.PKV.Q
No. 7	LA..C.S.I..LHINQ.AV.A..K....EA.........KAAL..E.QRI..ML.S.IQ....Q.S.Q.QDIQ.A.QAS.PKV.Q
No. 8	LA..C.S.I..LHINQ.AV.A..K....EA.........RAAL..E.QRI..ML.S.IQ....Q.S.Q.QDIQ.A.QAS.PKV.Q
HC-J8	LA..CIS.I..LH.ND.VV.A..K.I..EA.........KAAL..E.QRM..ML.S.IQ....Q..RQ.QDIQ.AIQ.S.PK..Q
MD2b1-2	LA..C.S.I...H.ND.AV.A..K....EA.........KAAL..E.QRM..ML.S.IQ....Q..RQ.QDIQ.AIQ.S.PK..Q
No. 9	LA..CIS.I..LH.ND.VV.T..K....EA.........KAAL..E.QRM..ML.S.IQ....Q..RQ.QDIQ.AIQ.S.PK..Q
No. 10	LA..CICSI...H.NDQVV.A..K....EA.........KAAL..E.QRM..ML.S.IQ....Q...Q.QDIQ.AIQ.S.PK..Q
No. 11	LA..CIS.I...H.ND.VV.A..K.I..EA.........KAAL..E.QRM..ML.S.IQ....Q..RQ.QDIQ.AIQ.S.PK..Q
No. 12	LA..CIS.I...H.NDHVV.A..K.I..EA.........KAAL..E.QRM..ML.S.IQ....Q..RQ.QDIQ.AIQ.S.PK..Q
No. 13	LA..CIS.I..LH.ND.VV.A..K.I..EA.........KAAL..E.QRM..ML.S.IQ....Q...Q.QDIQ.A.Q.S.PK..Q
No. 14	LA..CIS.I..LH.ND.VV.A..K.I..EA.........KAAL..E.QRM..ML.S.IQ....Q...Q.QDIQ.AIQ.S.PK..Q
No. 15	LA..CIS.I..LH.NDHVV.A..K.I..EA.........KAAL..E.QRM..ML.S.IQ....Q...Q.QDIQ.TIQ.S.PK..Q
No. 16	LA..CIS.I..VH.ND.VV.T..K.I..EA.........KAAL..E.QRI..ML.S.IQ....Q...Q.QDIQ.A.Q.S.PK..Q
No. 17	LA..CIS.I..LH.ND.VV.T..K.I..EA.........KAAL..E.QRI..ML.S.IQ....Q..RQ.QDIQ.AMQ.S.PK..Q

The same amino acids with group 1 peptide in each position are described as dots.

**Table:** (**B**)

No.	Genotype	Region 1	Region 2
Group 1 Peptide		RPAVIPDREVLYREFDEM	ECASHLPYIEQGMQLAEQF
1.	1b	K-----------Q----- (5)	------------------- (5)
2.	1b	----V------------- (5)	--T---------------- (5)
3.	1b	------------------ (3)	------------------- (5)
		--V--------------- (1)	
		---------------G-- (1)	
4.	1b	K----------------- (3)	------------------- (5)
		K-----------Q----- (2)	
Group 2 peptide		-AV-A--K----EA----	----RAAL--E-QRI--ML

The same amino acids with group 1 peptides are indicated by dashes. Group 2 peptides are also shown in the bottom line. The numbers of clones with the same sequence patterns are indicated in the parentheses. The amino acids that are the same as those of reference peptides of different genotypes are underlined.

**Table:** (**C**)

No.	Genotype	Region 1	Region 2
Group 2 Peptide		RAVVAPDKEVLYEAFDEM	ECASRAALIEEGQRIAEML
5.	2a	------------------ (5)	----K-------------- (4)
			---PK-------------- (1)
6.	2a	------------------ (5)	----K-------------- (4)
			----K---T---------- (1)
7.	2a	------------------ (5)	----K-------------- (4)
			------------------- (1)
8.	2a	------------------ (5)	------------------- (5)
9.	2b	-V--T------------- (4)	----K---------M---- (4)
		-I--T------------- (1)	--------------M---- (1)
10.	2b	QV---------------- (4)	----K---------M---- (4)
		QV-------I-------- (1)	----KV--------T---- (1)
11.	2b	-V-------I-------- (4)	----K---------M---- (5)
		-VA------I-------- (1)	
12.	2b	HV-------I-------- (4)	----K---------M---- (5)
		HV-M-----I-------- (1)	
13.	2b	-V-------I-------- (5)	----K---------M---- (5)
14.	2b	-V-------I-------- (5)	----K---------M---- (5)
15.	2b	HV-------I-------- (4)	----K---------M---- (5)
		HV-------I----S--- (1)	
16.	2b	-V--T----I-------- (5)	----K-------------- (3)
			----K-----K-------- (1)
			----K-D------------ (1)
17.	2b	-V--T----I-------- (4)	----K-------------- (5)
		-V--T----I-C------ (1)	
Group 1 peptide		-PA-I--R----RE----	----HLPY--Q-MQL--QF

The same amino acids with group 2 peptides are indicated by dashes. Group 1 peptides are also shown in the bottom line. The numbers of clones with the same sequence patterns are indicated in the parentheses. The amino acids that are the same as those of reference peptides of different genotypes are underlined.

**Table 6:** HCV genotypes by deep sequencing of HCV NS4 regions.

No.	H-77 (GT 1a)	HCV-J (GT 1b)	HC-J6 (GT 2a)	HC-J8 (GT 2b)	NZL1 (GT 3a), ED43 (GT 4a), EUH1480 (GT 5a), EUHK2 (GT 6a), QC69 (GT 7a)	Total log10 Reads
1.	<0.5%	99.40%	<0.5%	<0.5%	<0.5%	4.9
2.	<0.5%	99.52%	<0.5%	<0.5%	<0.5%	4.9
3.	<0.5%	99.49%	<0.5%	<0.5%	<0.5%	4.9
4.	<0.5%	99.87%	<0.5%	<0.5%	<0.5%	4.7
5.	<0.5%	<0.5%	99.41%	0.58% *	<0.5%	4.9
6.	<0.5%	<0.5%	99.34%	<0.5%	<0.5%	4.8
7.	<0.5%	<0.5%	99.92%	<0.5%	<0.5%	4.7
8.	<0.5%	<0.5%	99.87%	<0.5%	<0.5%	4.7
9.	<0.5%	<0.5%	<0.5%	99.55%	<0.5%	4.9
10.	<0.5%	<0.5%	<0.5%	99.87%	<0.5%	4.9
11.	<0.5%	<0.5%	<0.5%	99.78%	<0.5%	4.9
12.	<0.5%	<0.5%	<0.5%	99.80%	<0.5%	4.8
13.	<0.5%	<0.5%	0.57% *	99.26%	<0.5%	5.9
14.	<0.5%	<0.5%	<0.5%	99.51%	<0.5%	6.0
15.	<0.5%	<0.5%	<0.5%	99.57%	<0.5%	4.9
16.	<0.5%	<0.5%	<0.5%	99.26%	<0.5%	4.9
17.	<0.5%	<0.5%	<0.5%	99.32%	<0.5%	4.8
JFH-1	<0.5%	<0.5%	99.42%	<0.5%	<0.5%	6.0

GT, genotype; * Indicates minor populations.

**Table 7:** Treatment outcomes with or without the direct-acting antiviral agents (DAAs) in the present study.

No.	Treatment with DAAs	Outcomes (with DAAs)	Previous Treatment (without DAAs)	Outcomes (without DAAs)
1.	Daclatasvir plus asunaprevir for 24 weeks	SVR	(+)	Null response
2.	Daclatasvir plus asunaprevir for 24 weeks	SVR	(+)	Relapse
3.	NA	NA	(+)	Null response
4.	NA	NA	(+)	Relapse
5.	Sofosbuvir plus ribavirin for 12 weeks	SVR	(−)	NA
6.	Sofosbuvir plus ribavirin for 12 weeks	SVR	(−)	NA
7.	Sofosbuvir plus ribavirin for 12 weeks	SVR	(−)	NA
8.	Sofosbuvir plus ribavirin for 12 weeks	SVR	(−)	NA
9.	Sofosbuvir plus ribavirin for 12 weeks	SVR	(+)	Relapse
10.	Telaprevir with peginterferon plus ribavirin	SVR	(−)	NA
11.	Sofosbuvir plus ribavirin for 12 weeks	SVR	(−)	NA
12.	Peginterferon plus ribavirin for 24 weeks	SVR	(−)	NA
13.	NA	NA	(−)	NA
14.	NA	NA	(−)	NA
15.	NA	NA	(−)	NA
16.	NA	NA	(+)	Null response
17.	Sofosbuvir plus ribavirin for 12 weeks	SVR	(−)	NA
18.	NA	NA	(−)	NA

SVR, sustained virological response; NA, not available; (−), not performed; (+), performed.

## Abbreviations

HCV	Hepatitis C virus
NS	Nonstructural
5′-UTR	5′-untranslated region
RNA	Ribonucleic acid
PCR	Polymerase chain reaction
EIA	Enzyme immunoassay
IVDU	Intravenous drug user
ALT	Alanine transaminase
PLT	Platelet counts
ALB	Albumin
AFP	α-fetoprotein
GT	Genotype
SVR	Sustained virological response
HBsAg	Hepatitis B surface antigen
Anti-HIV	HIV antibody
ELISA	Enzyme-linked immunosorbent assay
cDNA	Complementary deoxyribonucleic acid
